# Does Bangkok have a central role in the dengue dynamics of Thailand?

**DOI:** 10.1186/s13071-020-3892-y

**Published:** 2020-01-13

**Authors:** Zhiwei Xu, Hilary Bambrick, Puntani Pongsumpun, I. Ming Tang, Laith Yakob, Gregor Devine, Francesca D. Frentiu, Gail Williams, Wenbiao Hu

**Affiliations:** 1 School of Public Health and Social Work, Queensland University of Technology, Brisbane, 4059 Australia; 2 Institute of Health and Biomedical Innovation, Queensland University of Technology, Brisbane, 4059 Australia; 3 School of Public Health, Faculty of Medicine, University of Queensland, Brisbane, 4006 Australia; 4 Department of Mathematics, Faculty of Science, King Mongkutʼs Institute of Technology Ladkrabang, Bangkok, 10520 Thailand; 5 Computational & Applied Science for Smart Innovation Cluster (CLASSIC), Faculty of Science, King Mongkutʼs University of Technology Thonburi, Bangkok, 10140 Thailand; 6 Department of Disease Control, London School of Hygiene and Tropical Medicine, London, WC1H 9SH UK; 7 Mosquito Control Laboratory, QIMR Berghofer Medical Research Institute, Brisbane, 4006 Australia; 8 School of Biomedical Sciences, Queensland University of Technology, Brisbane, 4059 Australia

**Keywords:** Bangkok, Dengue, Nakhon Ratchasima, Thailand

## Abstract

**Background:**

Bangkok plays a central role in the commerce of Thailand. This study aimed to characterize the district-level spatial-temporal patterns of dengue in Thailand and explore if a dengue peak in Bangkok led the peaks of dengue in other Thai provinces.

**Methods:**

Monthly dengue data at district level in Thailand from January 2004 to December 2017 were obtained and used to assess the spatial and seasonal patterns of dengue in Thailand. As our seasonal decomposition and cross-correlation analyses showed that dengue in Bangkok peaked in November, which was a few months after the dengue peak in most other provinces, we used a time-series generalized linear model to explore if there was another province in which the dengue case number was most predictive of dengue case numbers in other Thai provinces.

**Results:**

The highest district-level annual dengue incidence rates (per 10,000) in the three time periods (i.e. 2004–2008, 2009–2013 and 2014–2017) were 58.08 (Samphanthawong), 85.93 (Mueang Krabi), and 66.60 (Mae Sariang), respectively. Dengue incidence rates in the western part of Northern Thailand, southern part of Central Thailand, southern part of Eastern Thailand, and Southern Thailand were higher than in other regions. Dengue in most districts of Thailand peaked in June, July or August, but dengue peaks in all districts of Bangkok occurred in November. The number of dengue cases in Nakhon Ratchasima was most predictive of the number of dengue cases in other provinces in Thailand by a one-month lag.

**Conclusions:**

Our results suggest that the dengue peak in Bangkok did not lead the peaks of dengue in other Thai provinces. Future research exploring how changes in socio-ecological factors (e.g. road network and climate factors) in Nakhon Ratchasima have affected the transmission of dengue in Thailand might shed some new light on the prevention and control of dengue.

## Background

Dengue poses a substantial burden on the healthcare system and households of Thailand [1, 2]. Understanding the spatial pattern of dengue in Thailand and identifying those areas with high incidence rates are essential for wise allocation of limited public health resources. Existing studies have mainly explored the spatial pattern of dengue in Thailand using province-level data [3] or assessed the spatiotemporal patterns of dengue in one Thai province using village-level data [4]. There is a lack of nationwide analysis to unveil the spatial pattern of dengue in Thailand at a high spatial resolution (e.g. district-level data).

Unfolding the seasonal pattern of dengue is essential for understanding the drivers behind the occurrence of dengue and for the identification of optimal timing for vector control. Dengue may transmit from those regions with early dengue peak to the surrounding regions through many pathways (e.g. human movement [5, 6] and vector movement [7]). Bangkok plays a central role in the commerce of Thailand and it is one of the transportation hubs in Thailand, possibly facilitating dengue to be transmitted from Bangkok to other Thai provinces.

This study used monthly district-level dengue data from January 2004 to December 2017 in Thailand and aimed to fulfill two research objectives: (i) to elucidate the spatial and seasonal patterns of dengue at district-level in Thailand; and (ii) to explore whether the peak of dengue in Bangkok led the peaks of dengue in other Thai provinces.

## Methods

### Research site

Thailand is located in Southeast Asia and has 76 provinces. Bangkok, the capital city of Thailand, is a special administrative area (SAA). The 76 provinces and Bangkok can be grouped into six subnational regions according to climate pattern (https://www.tmd.go.th/en/), and these subnational regions are Northern Thailand, Northeastern Thailand, Central Thailand, Eastern Thailand, Southern Thailand West Coast and Southern Thailand East Coast. Sometimes Southern Thailand West Coast and Southern Thailand East Coast can be grouped into one category: Southern Thailand. Our prior paper has presented the locations of these subnational regions [3]. The administrative division levels of Thailand are province, district, sub-district and village. All together Thailand has 928 districts, including the 50 districts in Bangkok.

### Data collection

Monthly dengue incidence data in 716 districts of Thailand from January 2004 to December 2017 were obtained from the Ministry of Public Health of Thailand. In Thailand, doctors from the provincial level down to the village level are required to report the incidence of five to ten diseases (including dengue) to the Department of Epidemiology, Ministry of Public Health. Most of these doctors work in government hospitals or public health clinics. The districts in the present study were chosen because they had complete dengue data from January 2004 to December 2017. These districts covered all provinces except for Bueng Kan. Additional file 1: Figure S1 shows the locations of these 716 districts. We listed the districts by the latitude of the province which the districts belong to and gave each district a number for subsequent analysis. These numbers are presented in Additional file 2: Table S1.

The spatial analyses were conducted in ArcGIS version 10.5 [8] and all other analyses were conducted in R package version 3.5.0 [9].

### Data analysis

#### The spatial and seasonal patterns of dengue in Thailand

As a dengue epidemic cycle normally lasts for three to five years, we grouped the 14-year study period into three time periods in the spatial analysis, 2004 to 2008, 2009 to 2013 and 2014 to 2017. Average annual incidence of dengue of each district in each time period was calculated, and we used the average annual incidence data in the 716 districts to extrapolate the annual incidences of dengue in all 928 districts of Thailand [10]. The original spatial pattern (unsmoothed) of the average annual incidence of dengue in the 716 districts in each time period was also presented in the results.

We plotted the seasonal pattern of dengue in the 716 districts by means of a heat map. We also aggregated the district-level data into province-level data and plotted the seasonal pattern of dengue in the 75 provinces and Bangkok in a heat map.

#### Testing if the dengue peak in Bangkok led the peaks of dengue in other Thai provinces

Cross-correlation can show the correlation between two series (e.g. monthly dengue cases in Bangkok and monthly dengue cases in Chiang Mai) across different lags [11]. Using the aggregated province-level dengue data, we calculated the cross-correlation coefficients between Bangkok and all other provinces, and found that dengue peaks in Bangkok occurred few months after the dengue peaks in other provinces (details shown in the Results section). Hence, we used a generalized linear model with Poisson family (log link) to explore which province’s dengue case number was most predictive of other provinces’ dengue case numbers at one-month lag. The one-month lag was used because dengue transmission within a country, especially the transmission related to human movement, normally occurs in a few weeks [12, 13]. For example, we used the number of monthly dengue in Bangkok as dependent variable, and used the number of monthly dengue in Chiang Mai as independent variable, and controlled for seasonality and long-term trend to build up the model for Bangkok and Chiang Mai. Specifically, seasonality and long-term trend were controlled for through including “month” and “year” as dummy variables in the model. Due to the unavailability of province-level population data, we were unable to include the log scale of population in each year as an offset in the model. We added up the populations in all available districts in each province and used this as a proxy of the population in each province, and found including population in log-scale in the model as an offset did not change the results. Akaikeʼs information criterion (AIC) value and *R*^2^ value of the regression models were used to judge which province’s dengue case number was most predictive of other provinces’ dengue case numbers. Apart from a one-month lag, a two-month lag was also tested in the regression model and we found that one-month lag corresponded to a lower AIC value.

## Results

**Fig. 1:**
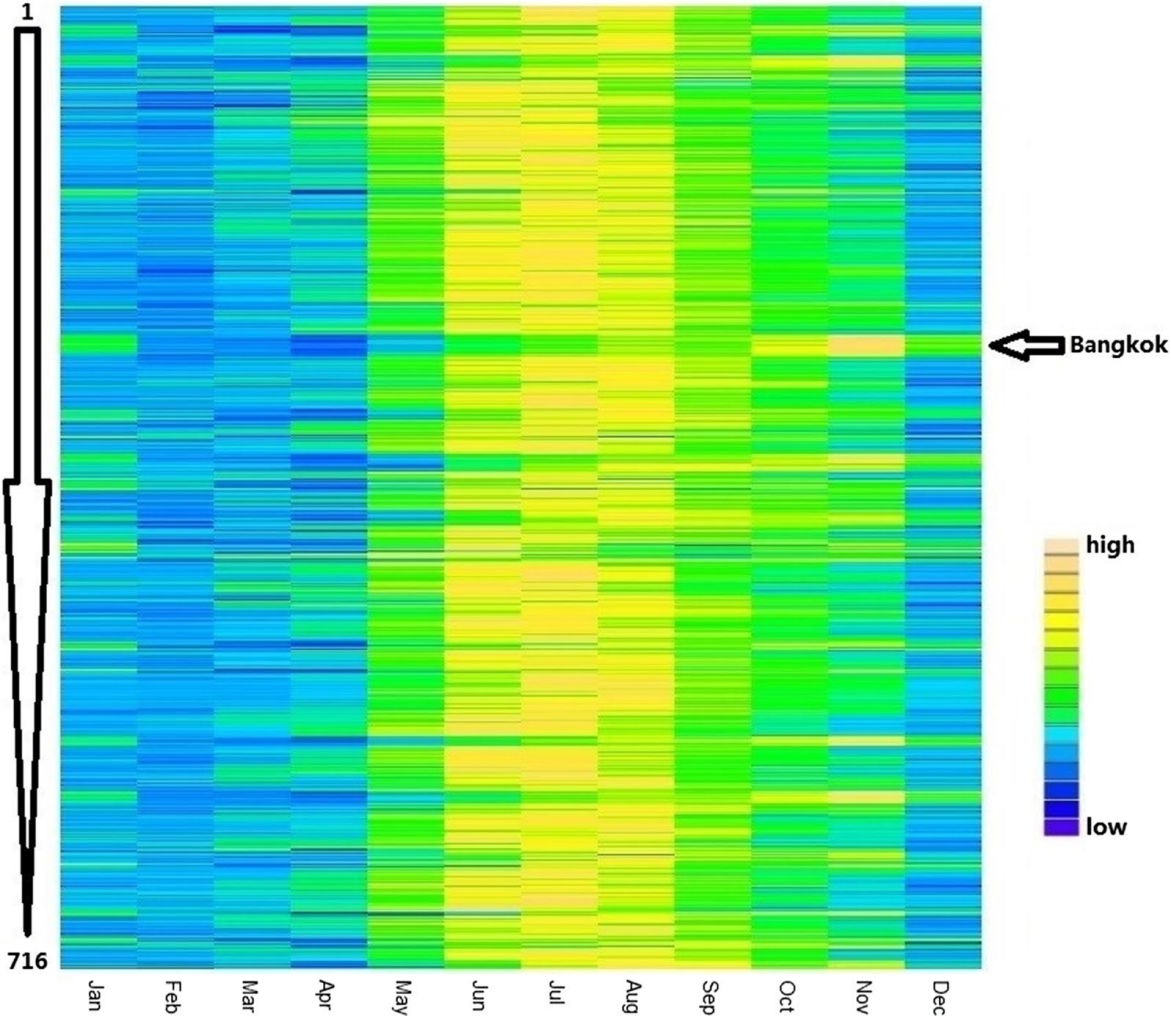
Seasonality of dengue in all selected districts of Thailand. “High” means a relatively higher number of dengue cases and “low” means a relatively lower number of dengue cases

Figure 1 illustrates the seasonal pattern of dengue in all selected districts. These districts were listed by latitude of the provinces which the districts belong to (high latitude to low latitude from the top to the bottom). Dengue peaks in the majority of these districts occurred in June, July or August. By contrast, dengue peaks in all districts of Bangkok occurred in November. The specific seasonal pattern of dengue at province-level (listed by latitude) is presented in Additional file 1: Figure S2a, and intriguingly, provinces with dengue peak being in October or November were located close to each other (Bangkok, Phra Nakhon Si Ayutthaya, and Samut Sakhon) (Additional file 1: Figure S2b).

**Fig. 2:**
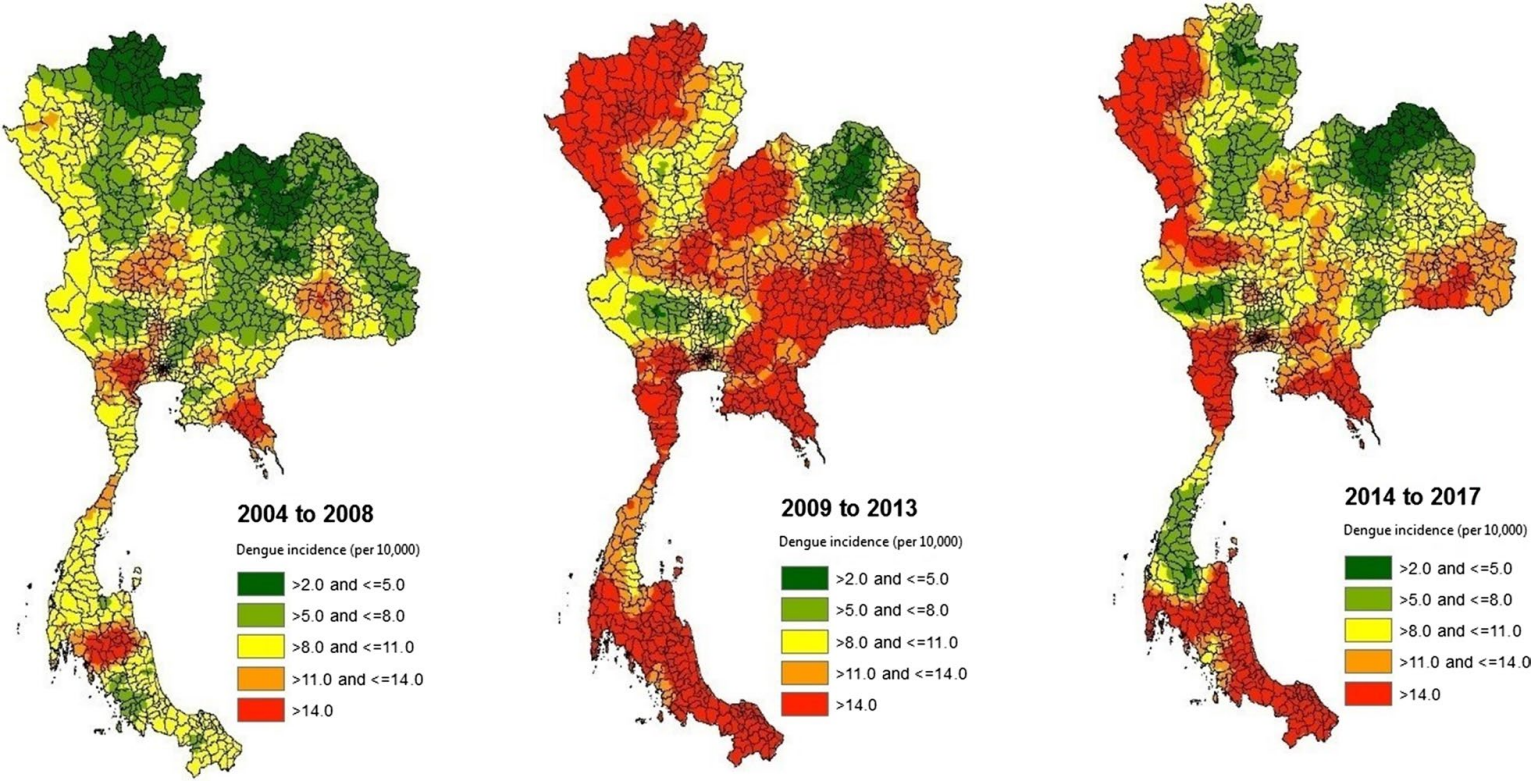
Spatial patterns of dengue in all districts of Thailand during three time periods. The figures were created using ArcGIS version 10.5 (ESRI Inc. Redlands, CA, USA)

Figure 2 shows the spatial patterns of dengue incidence rate in all districts of Thailand during three time periods. The unsmoothed spatial pattern of dengue incidence rate in the 716 districts of Thailand in each time period is presented in Additional file 1: Figure S3. The highest district-level dengue incidence rates (per 10,000) in the three time periods were 58.08 [Samphanthawong (Bangkok)], 85.93 [Mueang Krabi (Krabi)], and 66.60 [Mae Sariang (Mae Hong Son)], respectively. During the recent two time periods (i.e. 2009 to 2017), dengue incidence rates in the western part of Northern Thailand, southern part of Central Thailand, southern part of Eastern Thailand, and Southern Thailand were higher than in other regions. Dengue incidence rates in three provinces (i.e. Chanthaburi, Krabi and Samut Songkhram) were consistently high during three time periods. Our detailed analysis (results not shown) revealed that dengue incidence rate in Chanthaburi had been consistently high in all years from 2004 to 2017.

**Fig. 3:**
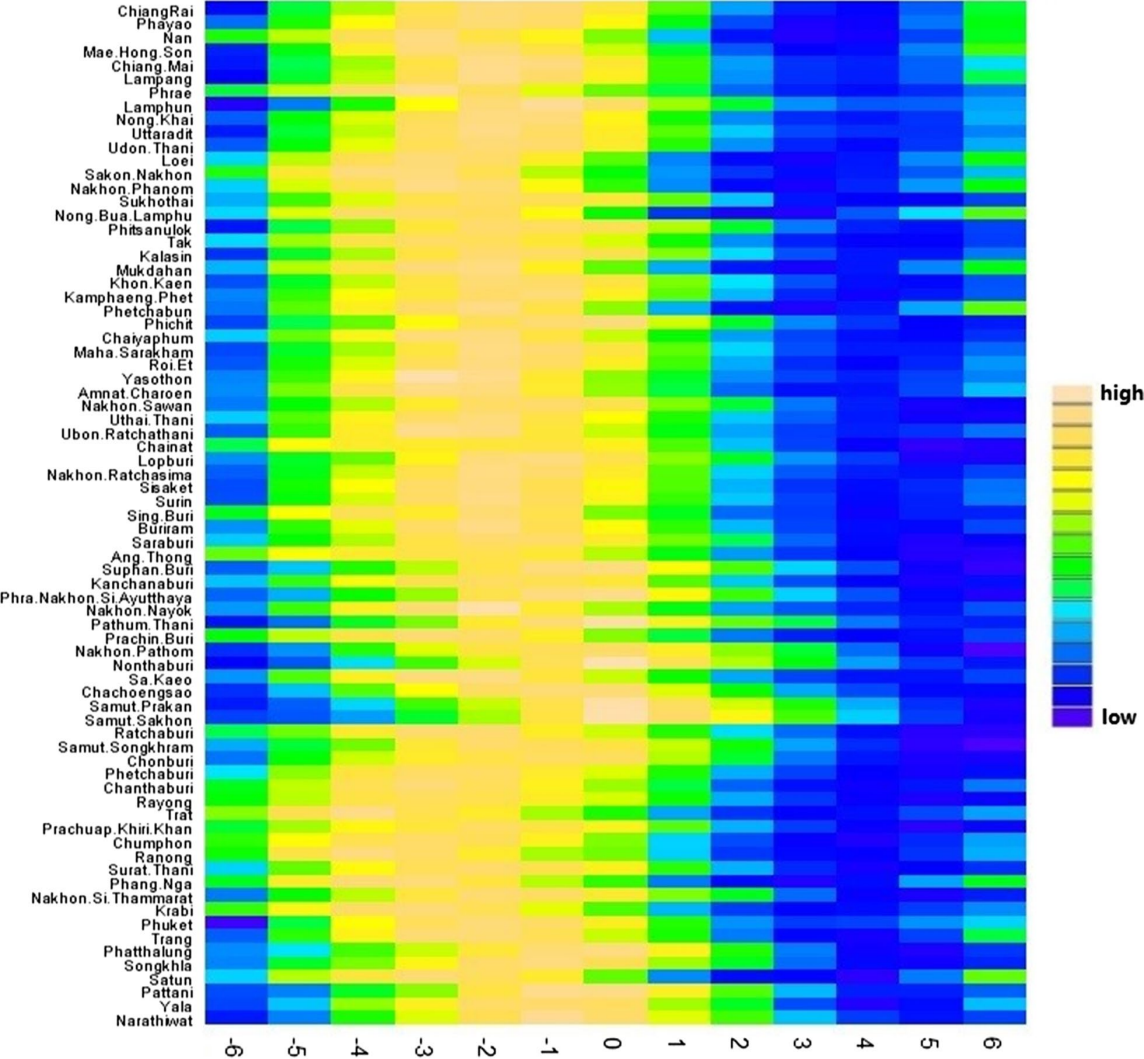
Cross-correlation between dengue in Bangkok and dengue in other provinces. “High” means a greater value of correlation coefficient and “low” means a lower value of correlation coefficient

Figure 3 shows the cross-correlation coefficients between dengue in Bangkok and dengue in all other provinces, suggesting that dengue peak in Bangkok was behind dengue peaks in most provinces.

**Fig. 4:**
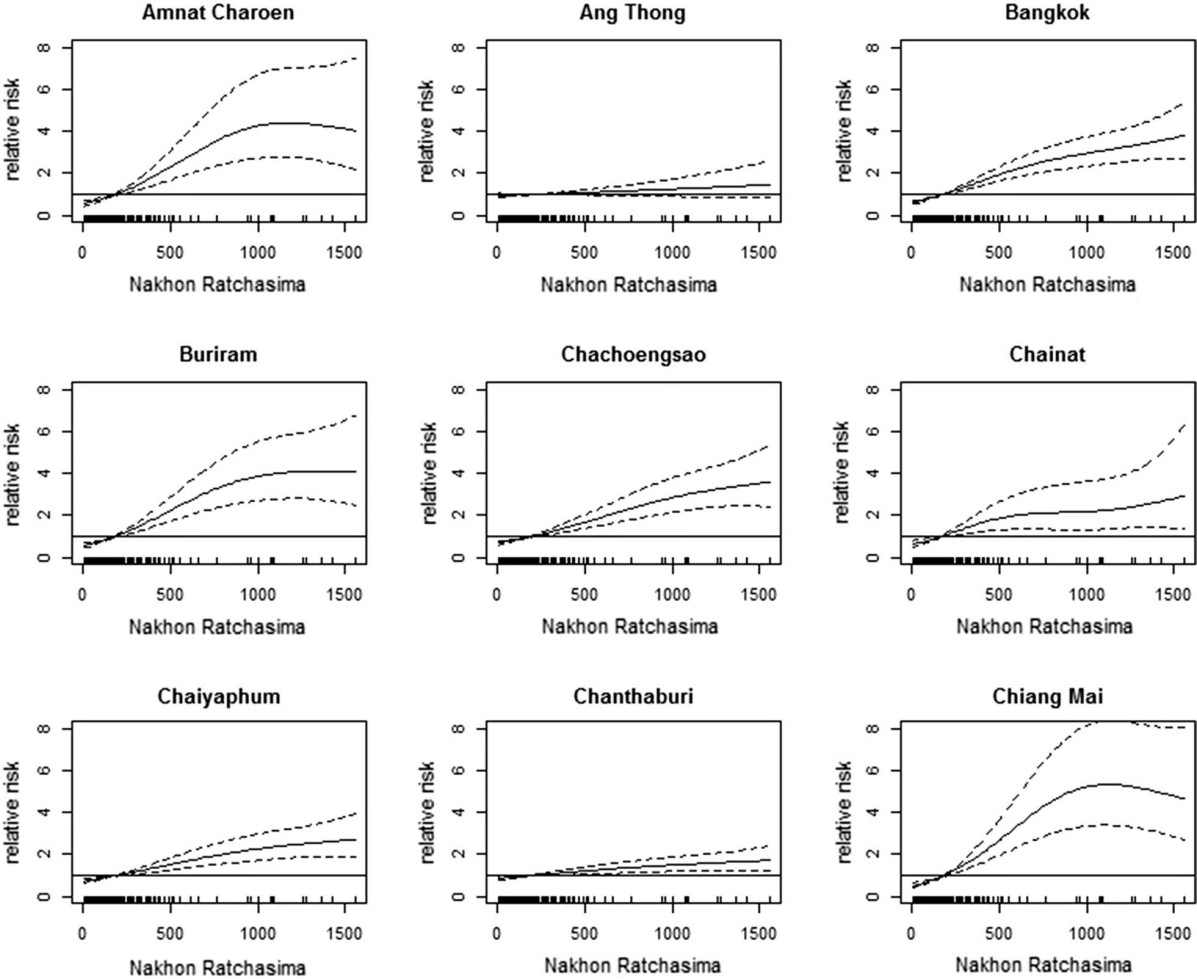
Association between dengue in Nakhon Ratchasima and dengue in other Thai provinces

Table 1 shows the values of AIC and mean values of R^2^ for the regression models of all provinces, clearly showing that the number of dengue cases in Nakhon Ratchasima performed the best in predicting the number of dengue cases in other provinces at one-month lag. In Table 1, the dependent variable for each model was not the total incidence in all of the other provinces combined, but each province being treated as a separate data point. Figure 4 and Additional file 1: Figure S4a–h present the plots of the Poisson regression models using the number of dengue cases in Nakhon Ratchasima to predict the number of dengue cases in other provinces at a one-month lag. The x-axis of these figures represents the monthly number of dengue cases in Nakhon Ratchasima.

**Table 1:** Model performance using dengue number of different provinces as the predictor

Province	AIC	Adj *R*^2^
Nakhon Ratchasima	589260.0	0.902
Sisaket	604370.2	0.866
Krabi	605194.3	0.776
Amnat Charoen	608963.5	0.851
Trat	614040.3	0.806
Prachin Buri	616421.4	0.872
Chanthaburi	621073.7	0.866
Rayong	627945.1	0.816
Ubon Ratchathani	629321.6	0.878
Chonburi	632763.4	0.859
Chaiyaphum	635303.7	0.842
Surin	636514.6	0.878
Sa Kaeo	644505.4	0.871
Buriram	647540.0	0.880
Pathum Thani	648675.8	0.883
Yasothon	650042.4	0.759
Loei	652182.9	0.769
Phang Nga	652279.8	0.799
Tak	653124.4	0.839
Saraburi	654334.2	0.831
Lopburi	655296.9	0.781
Lamphun	656718.1	0.827
Roi Et	658029.5	0.886
Chiang Mai	658371.0	0.827
Songkhla	660226.7	0.755
Nakhon Sawan	661585.7	0.871
Nakhon Phanom	661806.0	0.690
Mukdahan	663927.5	0.751
Phetchabun	664943.5	0.797
Phitsanulok	665121.1	0.886
Chainat	665735.7	0.769
Bangkok	667216.0	0.854
Uthai Thani	667821.3	0.821
Prachuap Khiri Khan	669487.2	0.801
Phuket	669929.6	0.808
Chachoengsao	672601.9	0.894
Udon Thani	672749.6	0.842
Khon Kaen	673944.0	0.897
Lampang	675347.7	0.764
Nakhon Pathom	675757.4	0.772
Kanchanaburi	676427.5	0.800
Trang	676589.4	0.689
Nong Bua Lamphu	677185.4	0.742
Maha Sarakham	677488.0	0.877
Samut Prakan	677599.0	0.832
Nakhon Nayok	678320.3	0.786
Phra Nakhon Si Ayatthaya	683286.5	0.872
Sakon Nakhon	684670.9	0.732
Nong Khai	688477.6	0.767
Ang Thong	689019.7	0.864
Surat Thani	690567.2	0.782
Chumphon	691803.1	0.720
Phrae	694140.0	0.771
Phayao	694980.6	0.752
Nakhon Si Thammarat	694986.5	0.754
Samut Sakhon	695688.3	0.893
Ratchaburi	697378.2	0.740
Sing Buri	699657.9	0.701
Nonthaburi	702054.9	0.842
Ranong	702380.7	0.724
Sukhothai	702870.5	0.847
Satun	704065.9	0.704
Samut Songkhram	705099.0	0.784
Chiang Rai	708097.7	0.726
Suphan Buri	710100.6	0.813
Phatthalung	711603.4	0.703
Mae Hong Son	711795.2	0.709
Narathiwat	712064.5	0.767
Kalasin	713185.1	0.851
Kamphaeng Phet	713376.7	0.776
Phetchaburi	715101.4	0.812
Uttaradit	715255.0	0.765
Yala	720502.4	0.712
Nan	725315.6	0.725
Pattani	726859.0	0.751
Phichit	727557.6	0.779

*Note*: A lower AIC value or/and a greater R^2^ value indicates a better model performance

*Abbreviation*: AIC, Akaikeʼs information criterion value; Adj *R*^2^, adjusted *R*^2^

## Discussion

This study, to the best of our knowledge, is the first nationwide study in Thailand which unveils the spatial and seasonal patterns of dengue at district-level, and is the first attempt to test if the dengue peak in Bangkok is leading the peaks of dengue in other Thai provinces. It has yielded three notable results. First, in the past decade, dengue incidence rates in western part of Northern Thailand, southern part of Central Thailand, southern part of Eastern Thailand, and Southern Thailand were higher than other regions. Secondly, dengue in most districts of Thailand peaked in June, July or August, but dengue peak in three provinces, including Bangkok, Phra Nakhon Si Ayutthaya, and Samut Sakhon, occurred in October or November, suggesting that the dengue peak in Bangkok did not lead the peaks of dengue in other Thai provinces. Thirdly, the number of dengue cases in Nakhon Ratchasima was most predictive of the number of dengue cases in other provinces at a one-month lag.

The dengue incidence rate during 2009–2013 was generally greater than it was during the other two time periods (i.e. 2004–2008 and 2014–2017), which can largely be attributable to the two dengue epidemic years (i.e. 2010 and 2013) [14]. Limkittikul et al. [15] have reported that Central Thailand was a region with high number of dengue cases and deaths from 2000 to 2011, and this region has a higher population density compared with other regions. In the present study, we observed that in the second and third time periods (2009 to 2017) southern part of Central Thailand had a high dengue incidence rate, indicating that this region may constitute a large proportion of Thailand dengue-related burden. Dengue incidence rate in the western part of Northern Thailand was not high in the first time period (i.e. 2004 to 2008), but it was consistently high in the second and third time periods. This region borders Myanmar and has been reported as a high-risk region for other vector-borne diseases (e.g. malaria) [16]. Although there was a temporal change in the spatial pattern of dengue incidence, we observed that dengue incidence rate in Chanthaburi, a province in Eastern Thailand, was consistently high across all years from 2004 to 2017. Available evidence in the literature on why dengue incidence rate in this province was consistently high is deficient, but it may be because Chanthaburi is next to a main river. Of note, Chanthaburi is the transport hub for accessing popular tourist spots (e.g. Koh Chang and Koh Kut), and its high dengue incidence rate may pose a threat to travelers.

Vector control remains the most viable option thus far for dengue control and prevention [17], and understanding the seasonality of dengue is of great value for identifying the optimal timing for intense vector control. In Thailand, all vector control programmes (e.g. in the country side or in the cities) are initiated by governments. Individuals are not allowed to initiate vector control projects without the permission of governments, therefore control programmes are applied consistently across jurisdictions. In this study, we found that dengue in Bangkok, Phra Nakhon Si Ayutthaya, and Samut Sakhon peaked in October or November, which is different from the June to August dengue peak pattern in most other provinces, suggesting a different optimal timing for intense vector control in these three provinces. The June to August dengue peak coincided with the rainy season in Thailand and this common peak was caused mainly by *Aedes aegypti*, and the October or November dengue peak might be caused by the arrival of the second mosquito species *Aedes albopictus* [18, 19].

Polwiang [20] has estimated the risk of dengue infection among travelers during their visit in Thailand from 2009 to 2015 and found that in general the risk of dengue infection from June to September was 2.50–4.07 times higher than it was from October to May. He has also observed that the risk of dengue infection in Chiang Mai was higher than Bangkok from May to September, but the risk of dengue infection in Chiang Mai was lower than Bangkok from October to April. Our finding that dengue in Bangkok peaked in November and Polwiang’s finding both suggested that future travelers visiting Thailand need to be given information on the specific dengue seasonal pattern of their destinations prior to their travel.

The present study using dengue data from 2004 to 2017 and our previous work using severe dengue data from 1999 to 2014 [3] found that dengue and severe dengue in Bangkok peaked in November, which was after the peak of most other provinces. This suggested that dengue peak in Bangkok occurred a few months after dengue peaks in other Thai provinces. As the economy of Thailand has developed over the past decades, Bangkok is no longer the only center of transportation networks. We found that the number of dengue cases in Nakhon Ratchasima performed exceptionally well in predicting the number of dengue cases in other provinces (including Bangkok) at one-month lag. Nakhon Ratchasima is the largest province by area in Thailand with a population of approximately 2.7 million, and it is the center of transportation in Northeastern Thailand. These characteristics may allow Nakhon Ratchasima to play a central role in the dengue dynamics of Thailand, although more in-depth studies need to be done prior to any concrete conclusions being made.

Human movement is one of the drivers of dengue transmission and it largely relies on public transport. Infected mosquitoes can easily be transported by trucks traveling on the road network. Most of the effort in Thailand towards developing a transportation network in Thailand has been on developing road networks [21]. For example, 77% of the governmental effort in the period 2006 to 2010 was devoted to improving the road transportation network while only 18% was directed towards improving the rail network. For the period 2011 to 2020, 46% will be devoted to the rail network while only 39% will be devoted to the road network [21]. Future studies looking at how road network and human movement around Nakhon Ratchasima affect the transmission of dengue may help unveil the reasons behind dengue transmission in Thailand. Moreover, the development of future dengue early warning system may also need to incorporate information on human movement.

This study is the first nationwide study in Thailand to explore the spatiotemporal patterns of dengue at district-level, with recent data. The seasonality of dengue in Bangkok, Phra Nakhon Si Ayutthaya, and Samut Sakhon that we observed can provide useful information for future vector control and for giving judicious advice to international travelers on the ideal timing of travelling. This study also suggested that the number of dengue cases in Nakhon Ratchasima was most predictive of the number of dengue cases in other Thai provinces, motivating future attempts to explore if Nakhon Ratchasima plays a central role in the dengue dynamics of Thailand. Three limitations of this study should also be acknowledged. First, due to data unavailability, we were only able to use data from 716 districts instead of all 928 districts, and we did not have any information on dengue in Bueng Kan. Secondly, we were unable to assess the district-level socioecological factors (e.g. human movements and climate, etc.) which impact the occurrence of dengue as the data were unavailable. Future research aiming to fill this data gap based on spatiotemporal model is warranted. Thirdly, due to the unavailability of province-level population data, we were unable to include the population in log scale as an offset in the regression model. Fourthly, similar to other Asian countries, under-reporting issues may exist for dengue national surveillance data in Thailand [15].

## Conclusions

Dengue incidence rates in western part of Northern Thailand, southern part of Central Thailand, southern part of Eastern Thailand, and Southern Thailand were higher than other regions of Thailand in the past decade. Although dengue peaked in June, July or August in most Thai provinces, three provinces (i.e. Bangkok, Phra Nakhon Si Ayutthaya and Samut Sakhon) had a dengue peak in October or November. Future endeavors aiming to unfold the transmission pattern of dengue in Thailand may need to pay more attention to Nakhon Ratchasima. Exploring how human movement in Thailand (especially around Nakhon Ratchasima) affects dengue transmission, and incorporating this information into the development of dengue early warning system may aid in dengue control and prevention.

## Supplementary information

**Additional file 1: Figure S1.** Locations of the selected 716 districts. **Figure S2. a** Seasonality of dengue in different provinces (listed by latitude). **b** Dengue peak month in different provinces. **Figure S3.** The original spatial pattern of annual dengue incidence rate in the 716 districts. **Figure S4. a**–**h** Association between dengue in Nakhon Ratchasima and dengue in other Thai provinces.

**Additional file 2: Table S1.** The names of the 716 districts included in this study and the provinces which these districts belong to.

## Data Availability

The data that support the findings of this study are available from the Ministry of Public Health of Thailand but restrictions apply to the availability of these data, and thus are not publicly available. However, data are available from the authors upon reasonable request and with the permission of the Ministry of Public Health of Thailand.

## Abbreviations

AIC: Akaikeʼs information criterion
SAA: special administrative area
