# Supplementary material for: Does Bangkok have a central role in the dengue dynamics of Thailand?
Source: Parasit Vectors. 2020 Jan 13;13:22. doi: 10.1186/s13071-020-3892-y (PMC6958813; doi:10.1186/s13071-020-3892-y)

Additional file 1

Figure S1. Locations of the selected 716 districts. This figure was created using ArcGIS version 10.5 (ESRI Inc. Redlands, CA, USA)

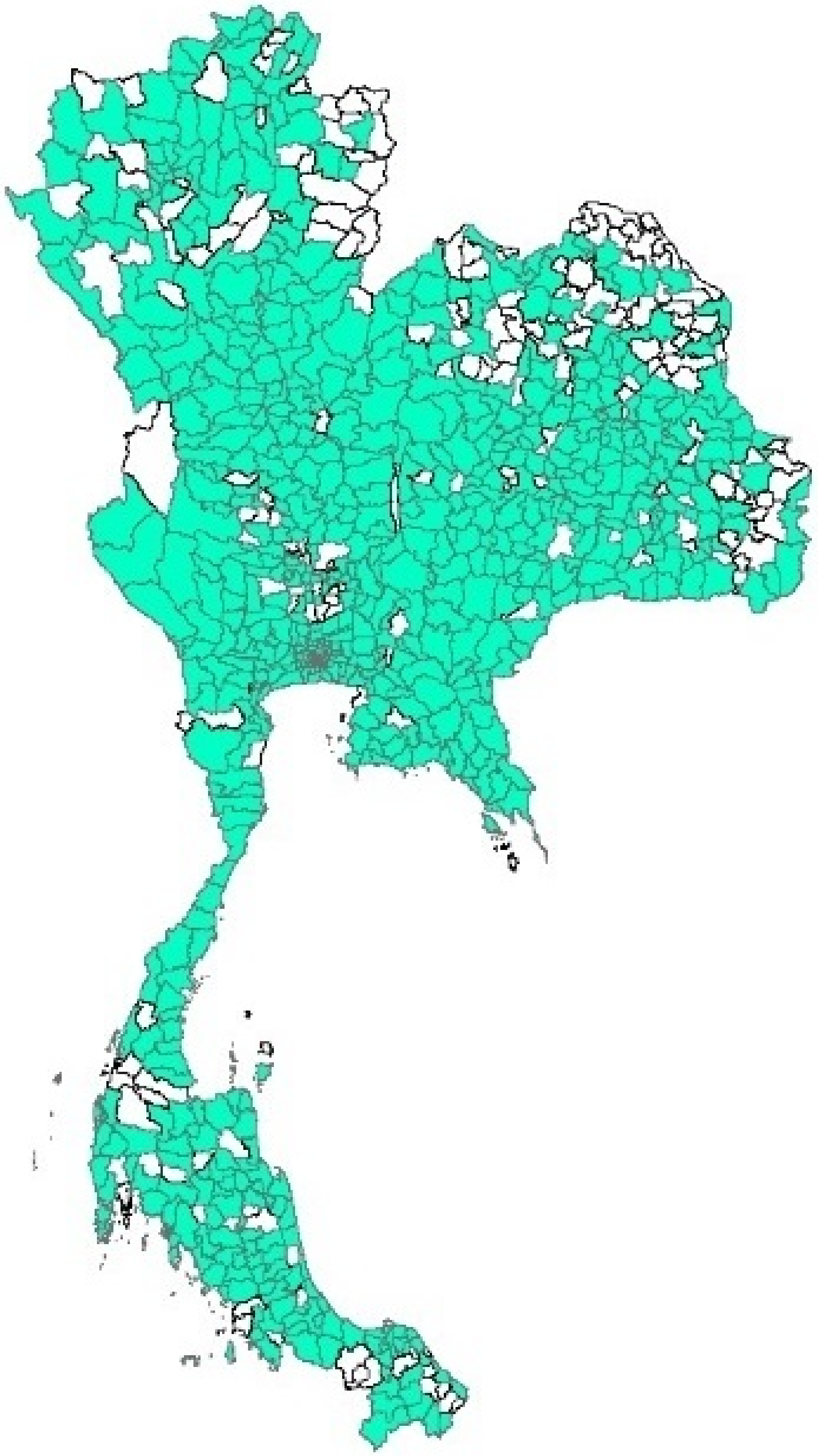

Figure S2A. Seasonality of dengue in different provinces (listed by latitude)

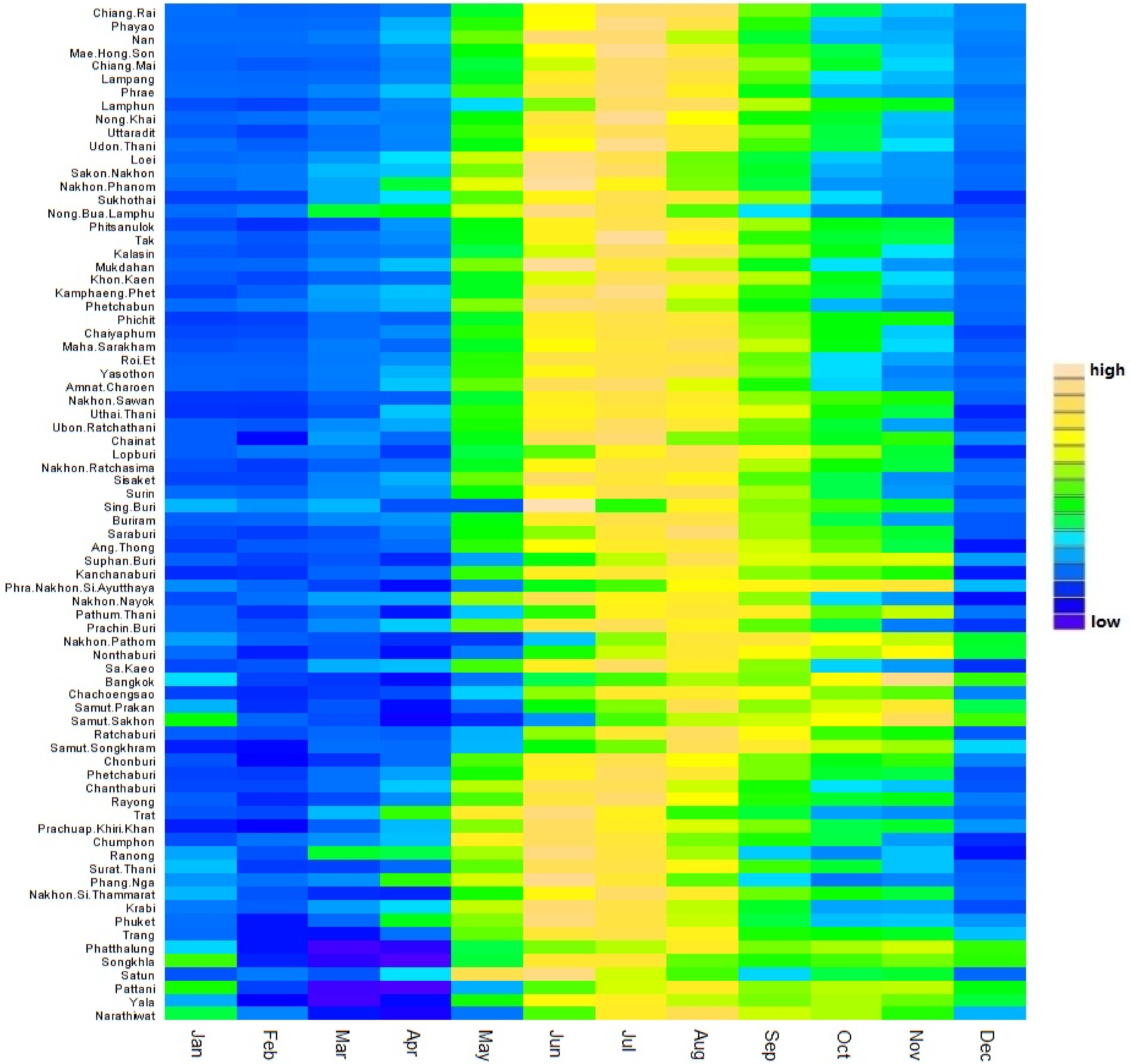

Figure S2B. Dengue peak month in different provinces. This figure was created using ArcGIS version 10.5 (ESRI Inc., Redlands, CA, USA)

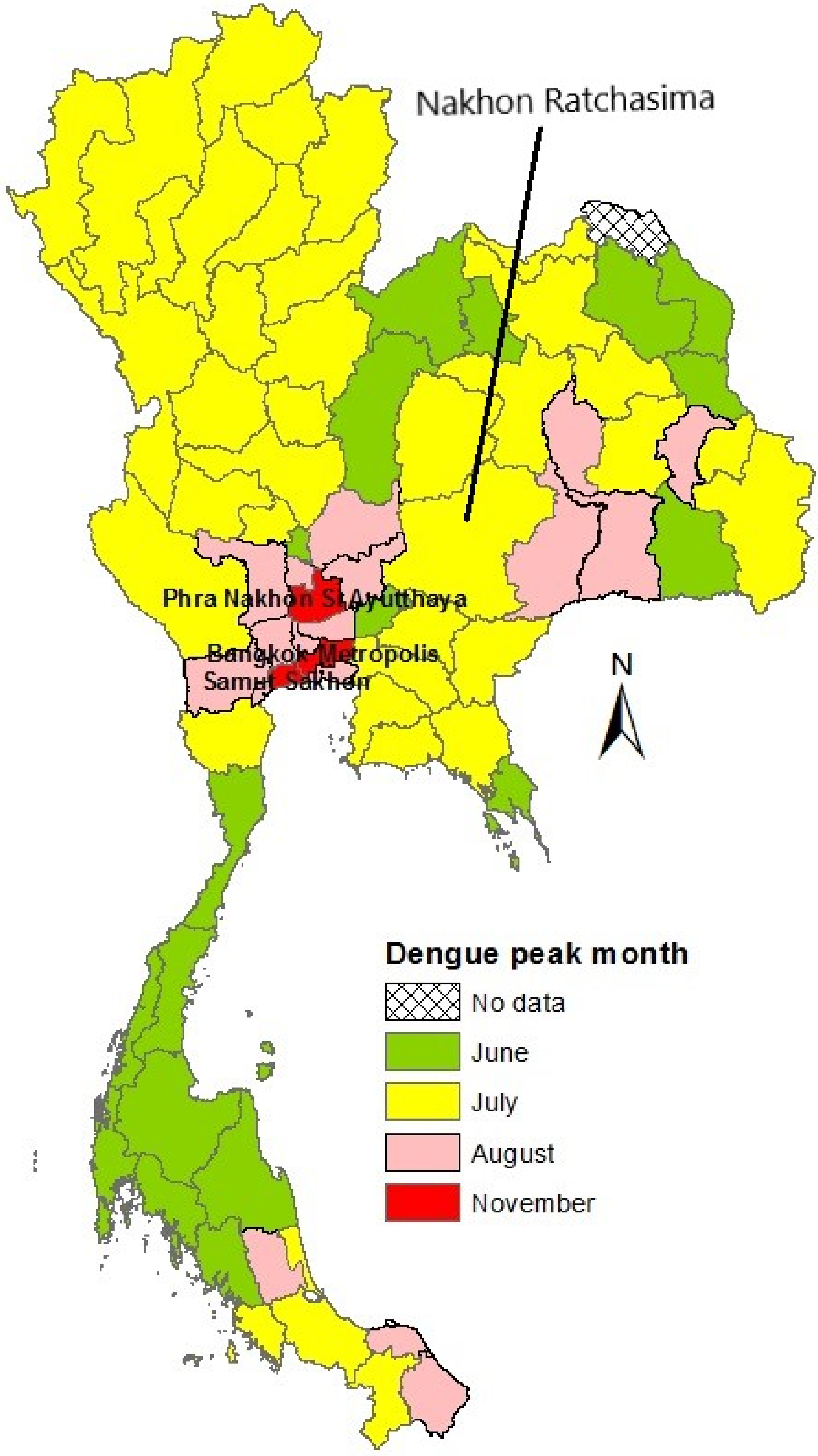

Figure S3. The unsmoothed patterns of dengue incidence in the 716 districts of Thailand during three time periods. The figures were created using ArcGIS version 10.5 (ESRI Inc. Redlands, CA, USA).

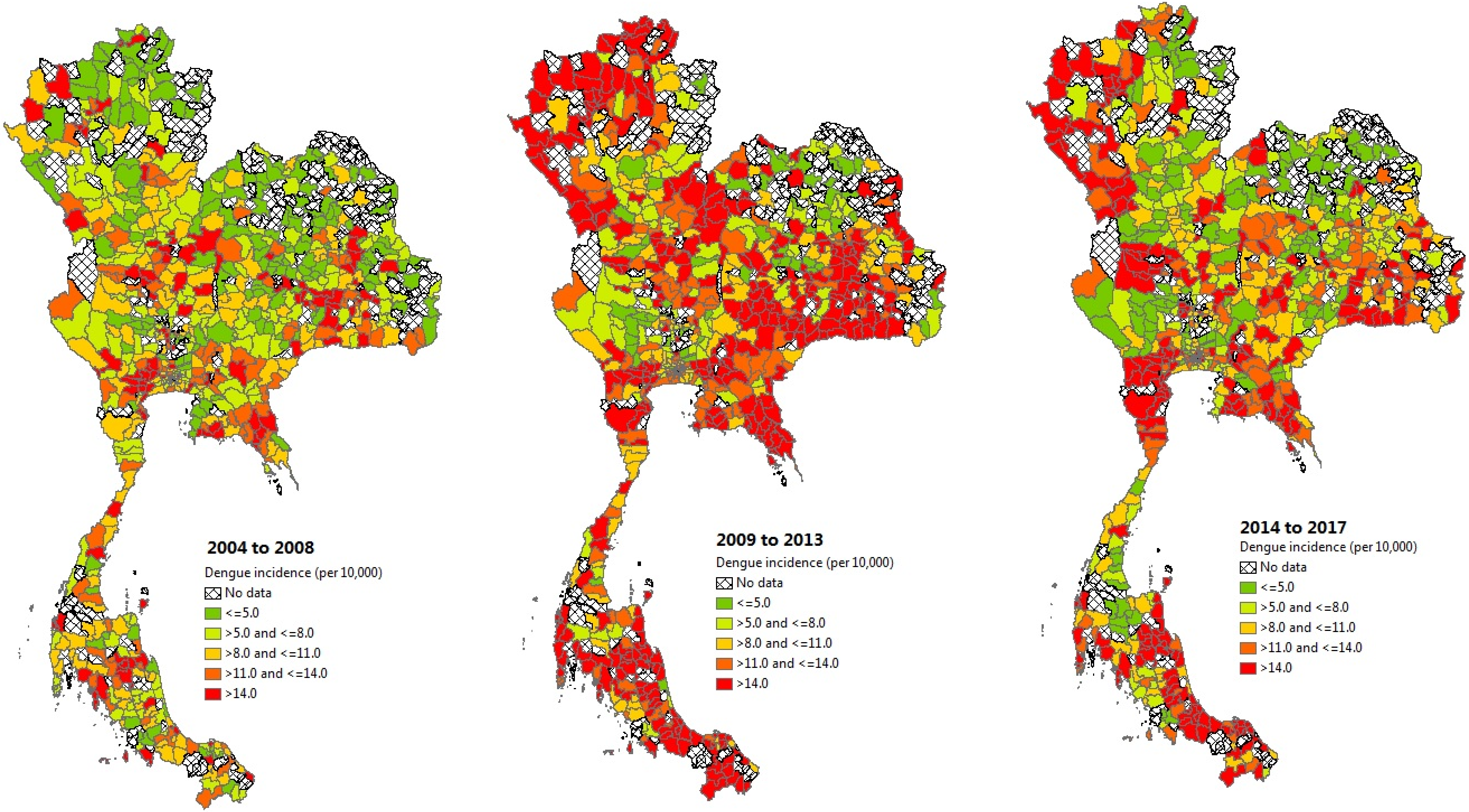

Figure S4A. Association between dengue in Nakhon Ratchasima and dengue in other Thai provinces

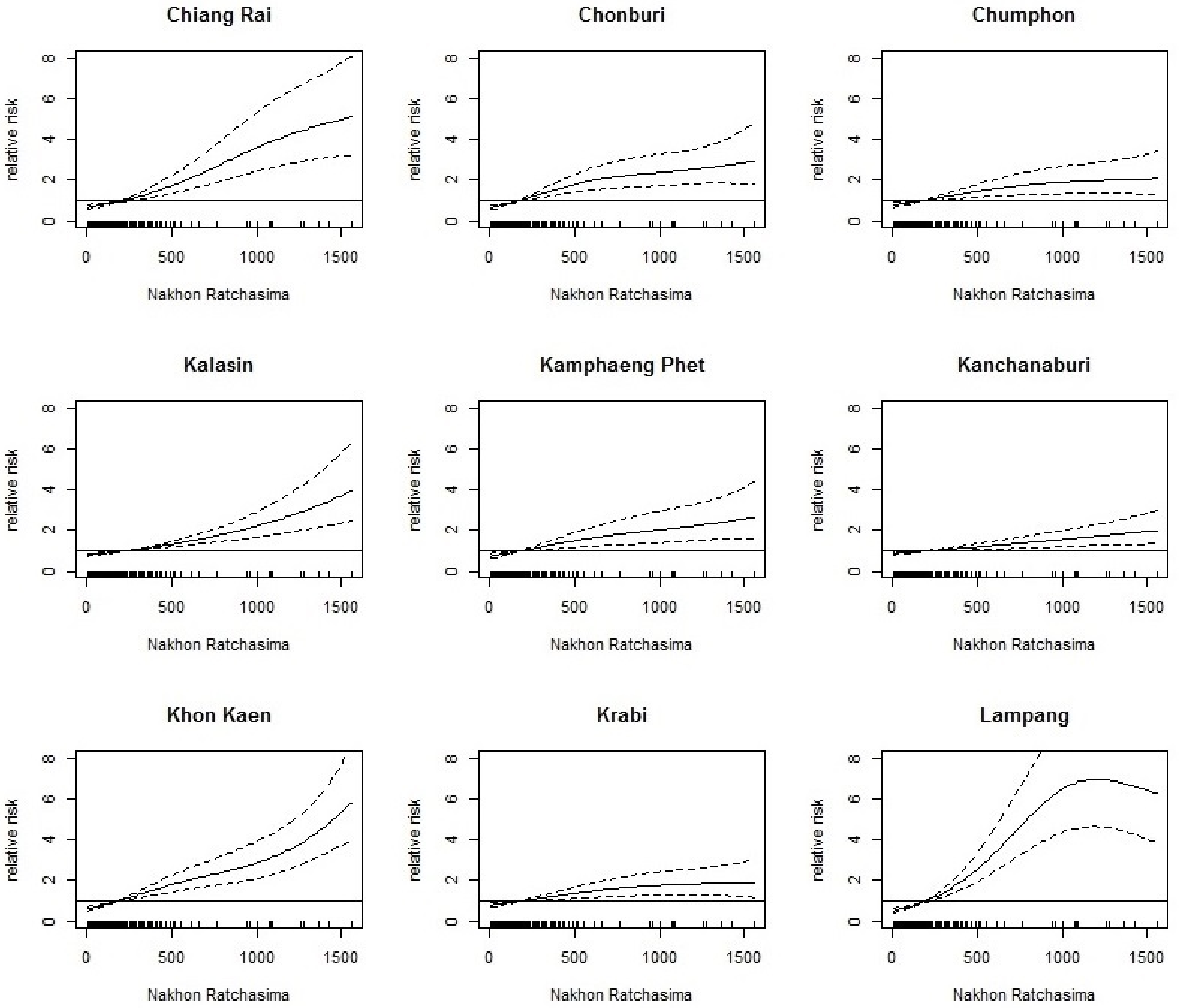

Figure S4B. Association between dengue in Nakhon Ratchasima and dengue in other Thai provinces

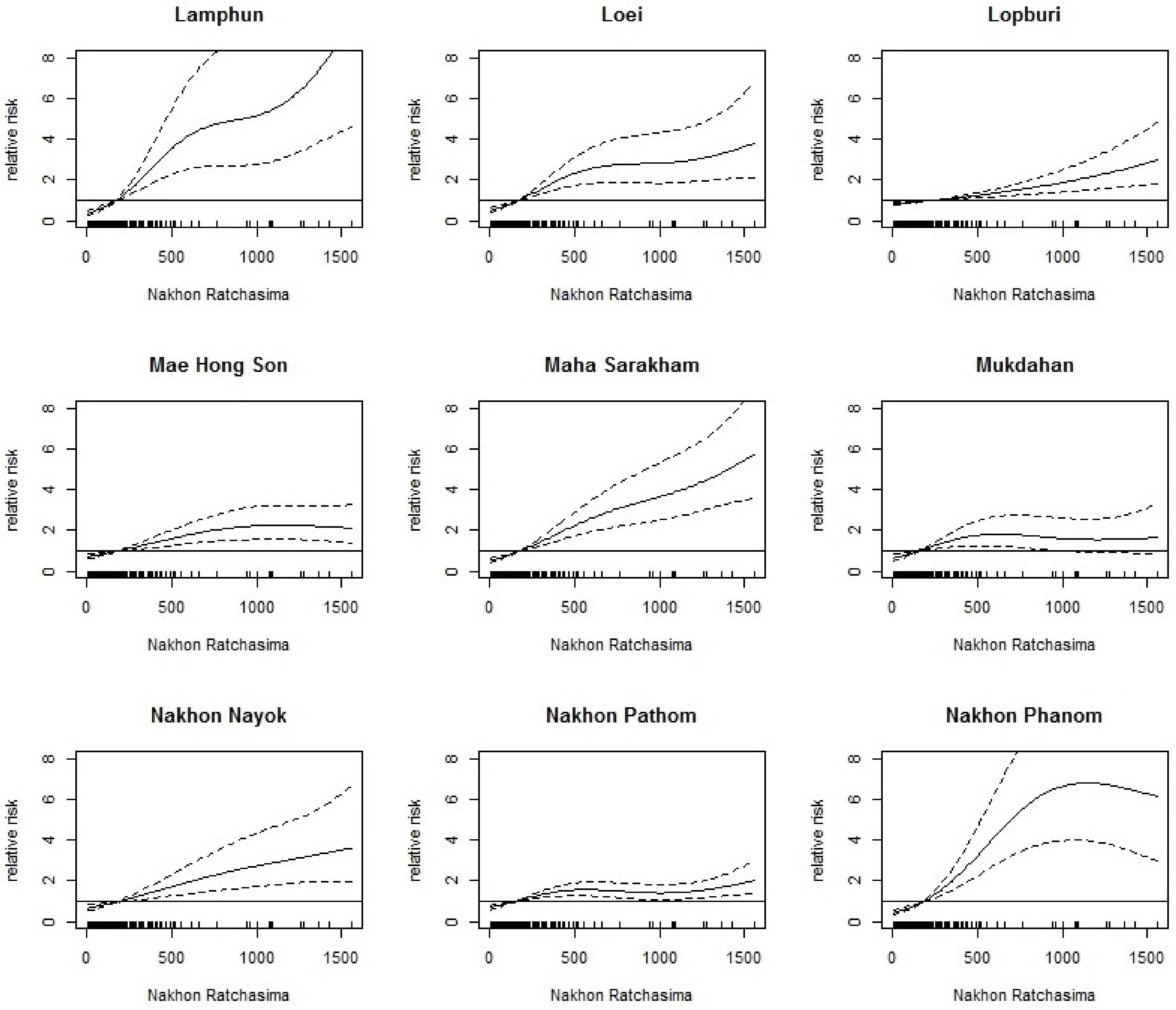

Figure S4C. Association between dengue in Nakhon Ratchasima and dengue in other Thai provinces

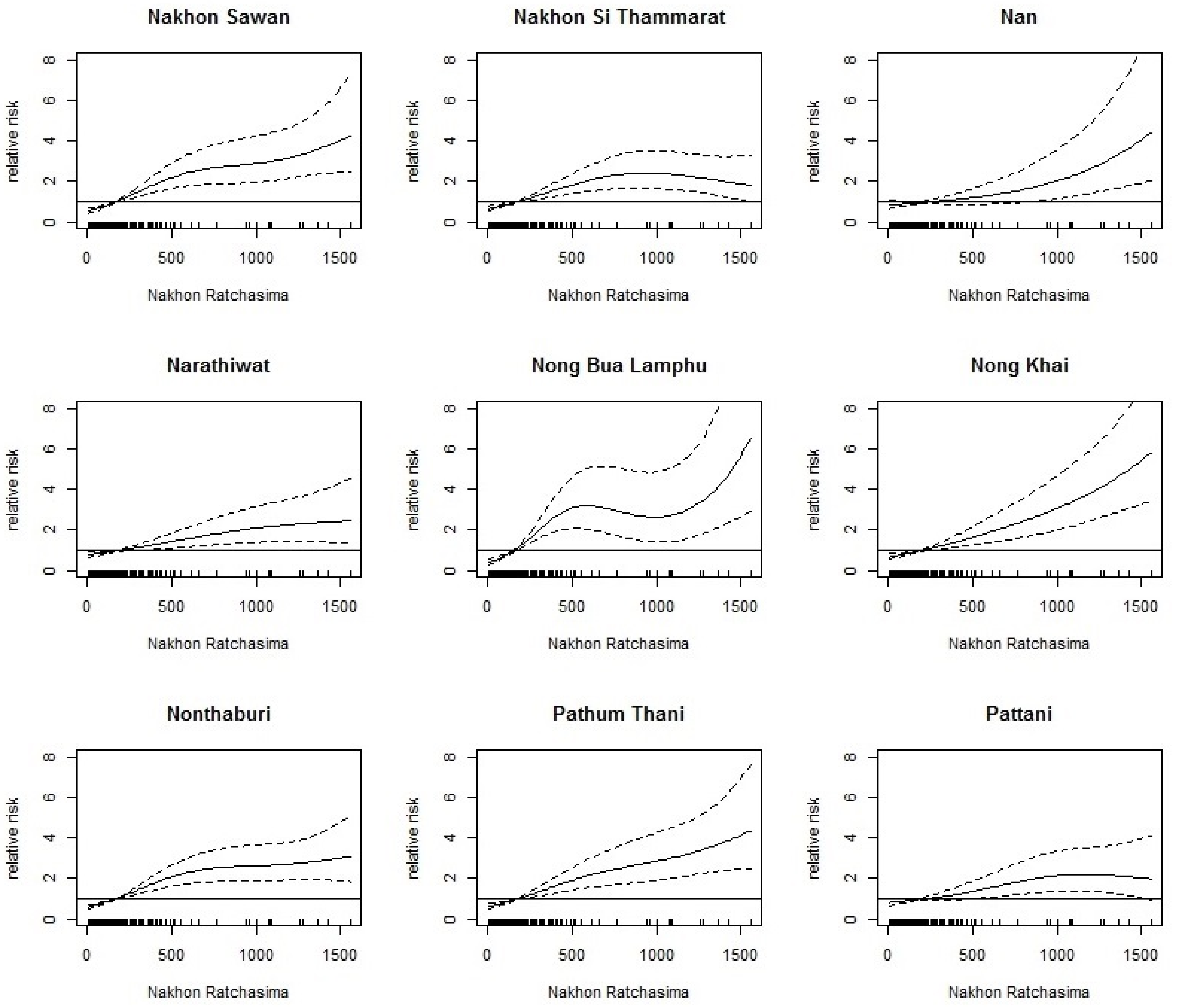

Figure S4D. Association between dengue in Nakhon Ratchasima and dengue in other Thai provinces

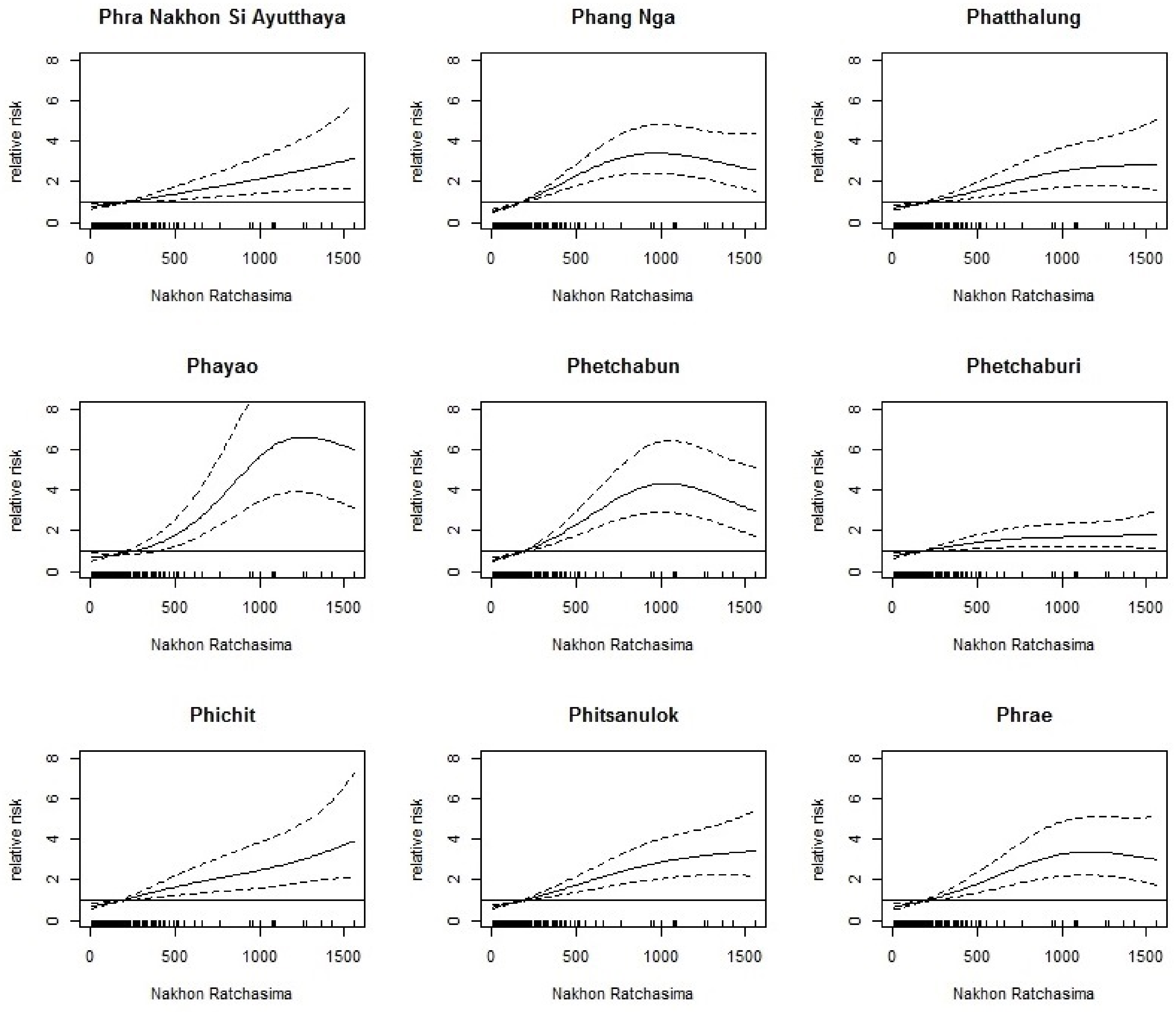

Figure S4E. Association between dengue in Nakhon Ratchasima and dengue in other Thai provinces

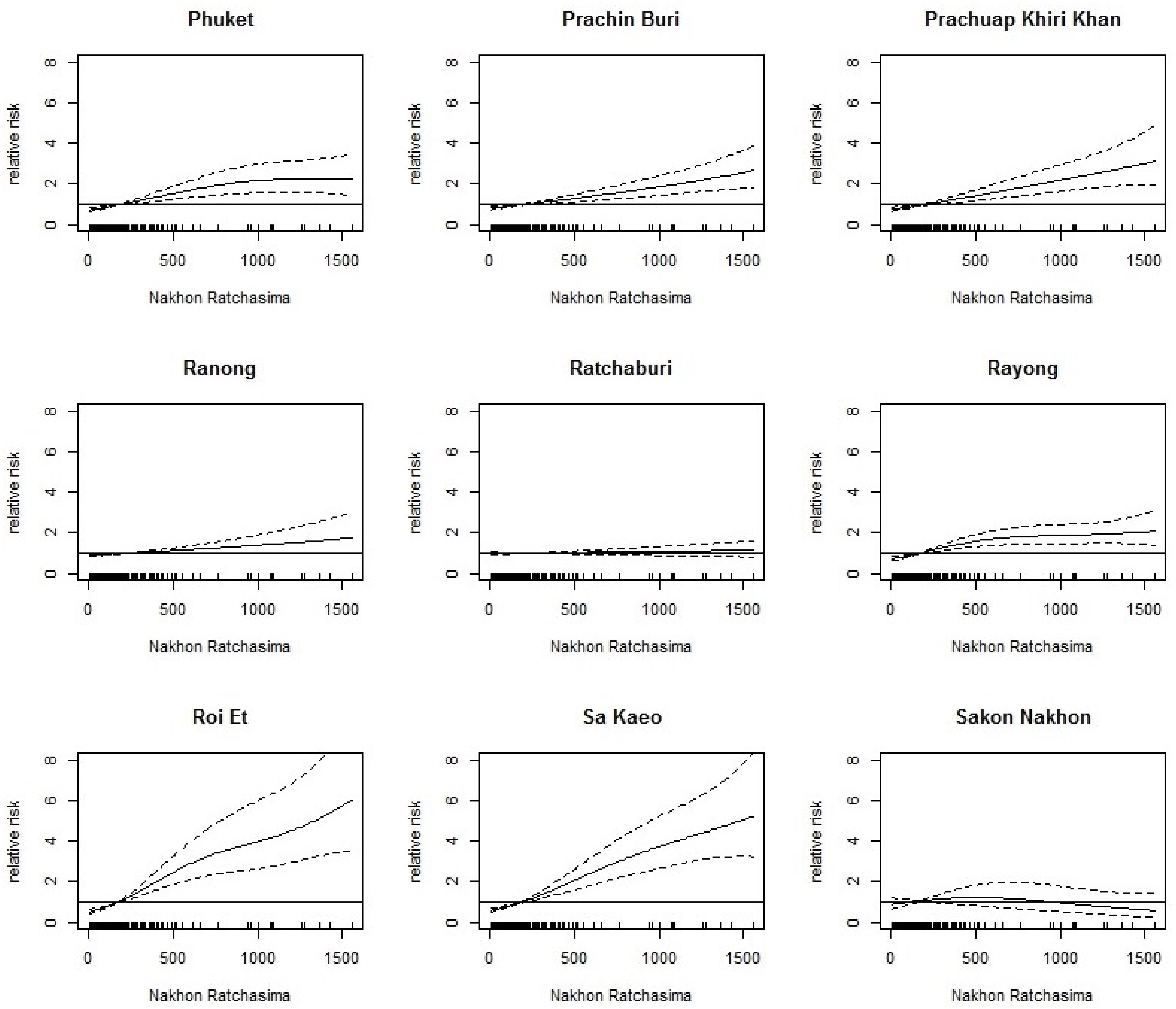

Figure S4F. Association between dengue in Nakhon Ratchasima and dengue in other Thai provinces

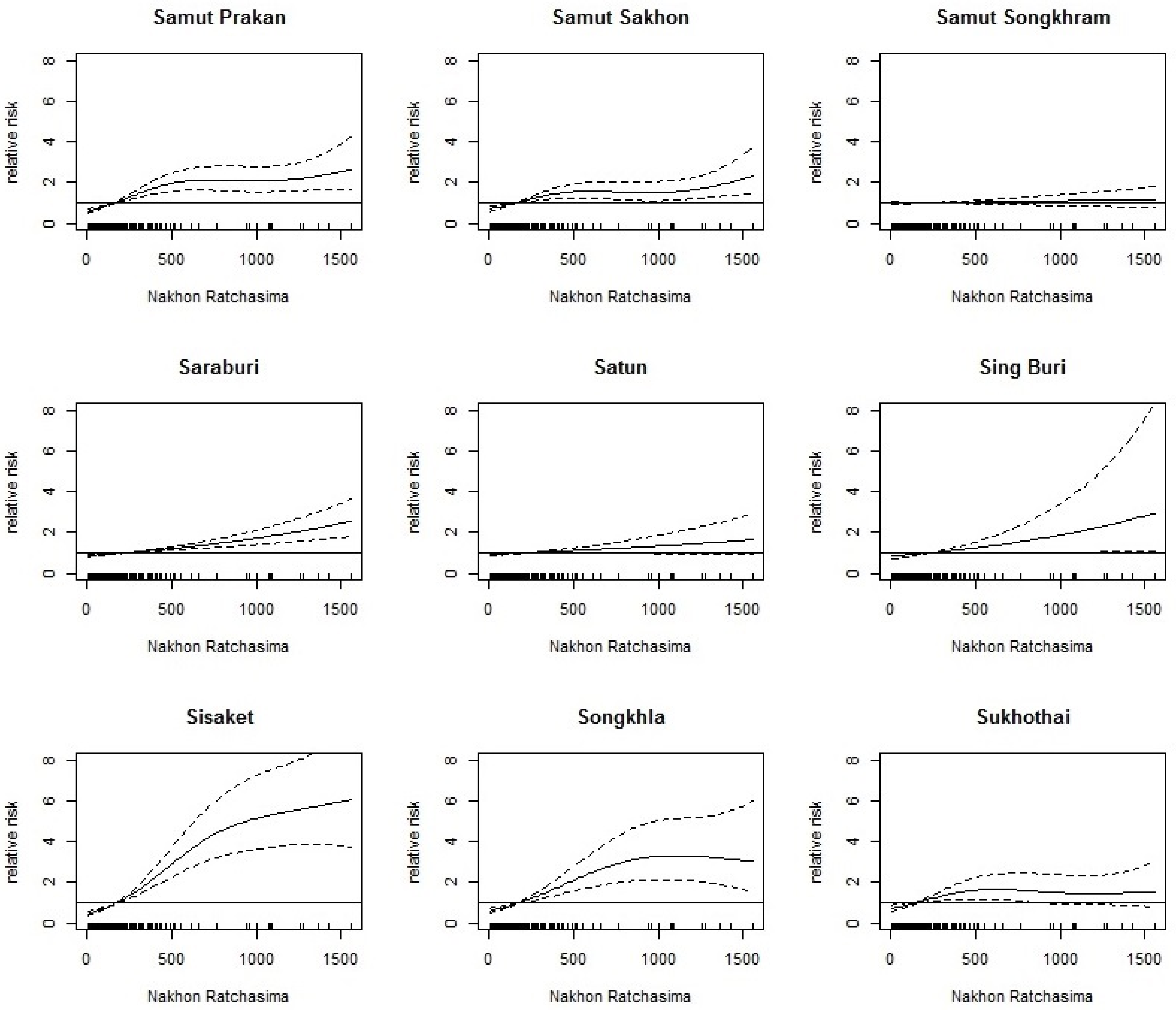

Figure S4G. Association between dengue in Nakhon Ratchasima and dengue in other Thai provinces

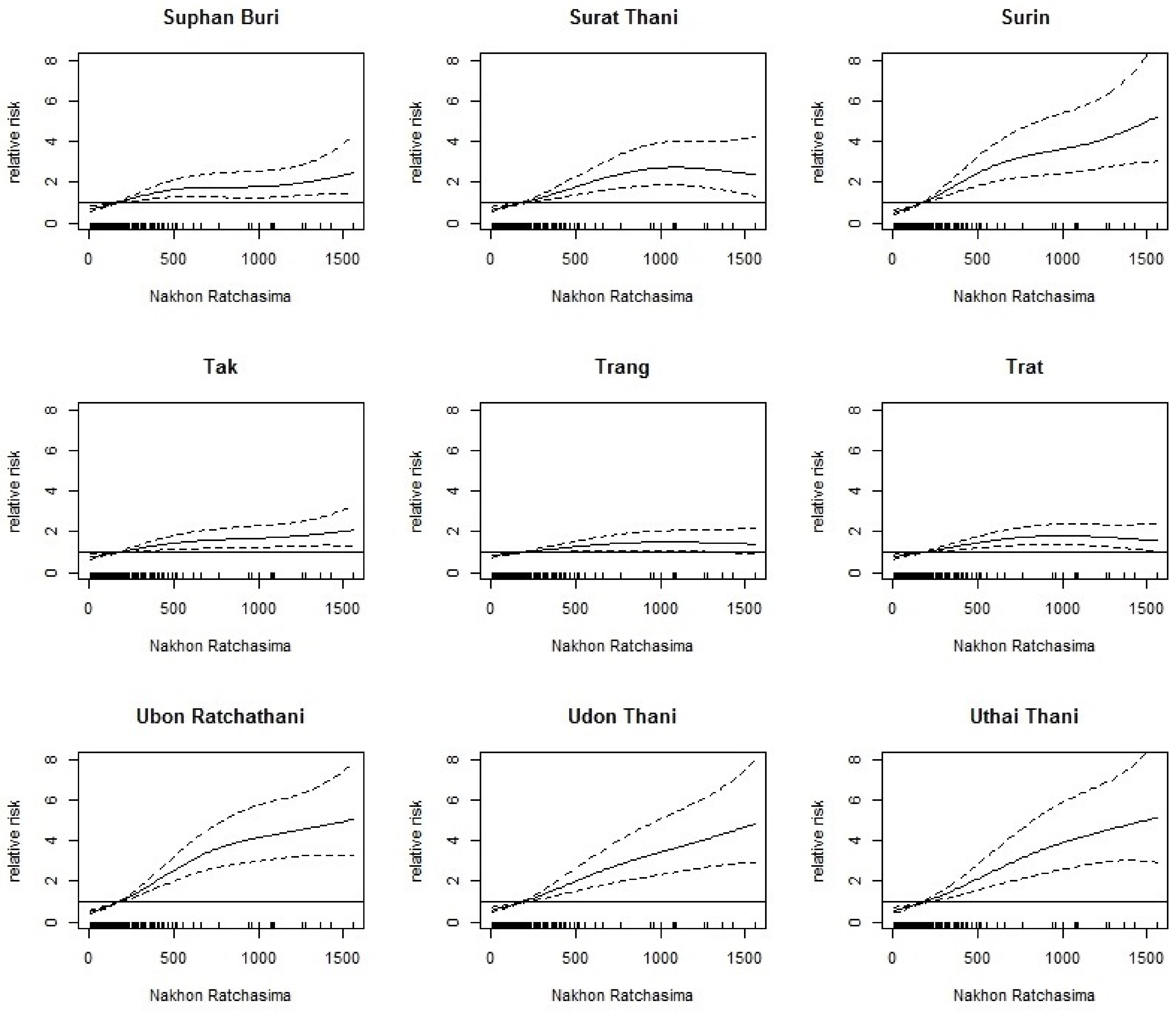

Figure S4H. Association between dengue in Nakhon Ratchasima and dengue in other Thai provinces

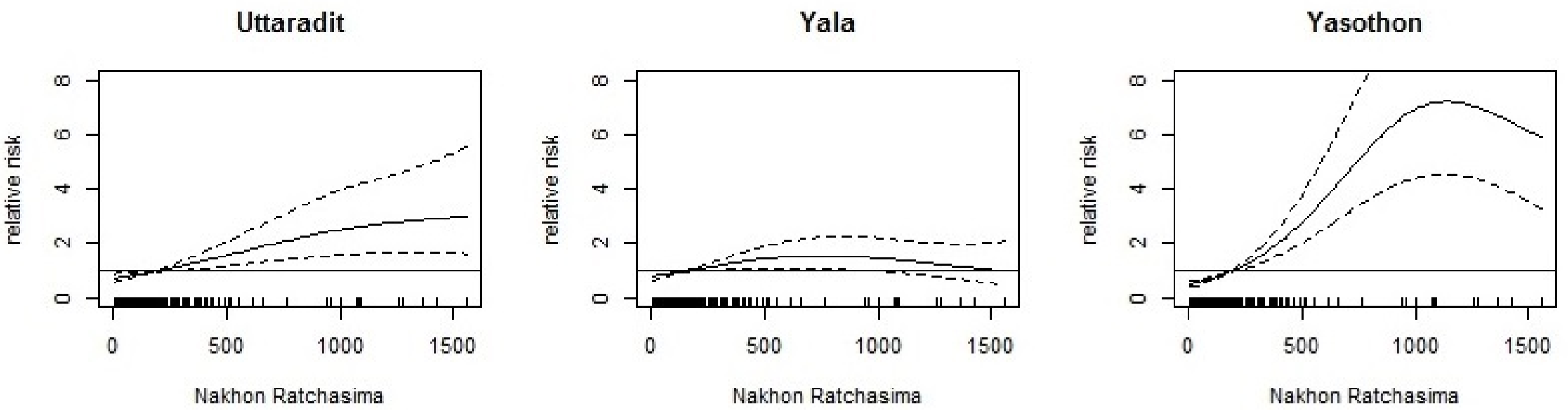

Supplement: Supplementary file 1 — Additional file 1: Figure S1. Locations of the selected 716 districts. Figure S2. a Seasonality of dengue in different provinces (listed by latitude). b Dengue peak month in different provinces. Figure S3. The original spatial pattern of annual dengue incidence rate in the 716 districts. Figure S4. a–h Association between dengue in Nakhon Ratchasima and dengue in other Thai provinces. [file 13071_2020_3892_MOESM1_ESM.pdf]
